# A systematic review of three approaches for constructing physical activity messages: What messages work and what improvements are needed?

**DOI:** 10.1186/1479-5868-7-36

**Published:** 2010-05-11

**Authors:** Amy E Latimer, Lawrence R Brawley, Rebecca L Bassett

**Affiliations:** 1 School of Kinesiology and Health Studies, Queen's University, Kingston, ON, Canada; 2 College of Kinesiology, University of Saskatchewan, Saskatoon, SK, Canada; 3 Department of Kinesiology, McMaster University, Hamilton, ON, Canada

## Abstract

**Background:**

To motivate individuals to adhere to a regular physical activity regime, guidelines must be supplemented with persuasive messages that are disseminated widely. While substantial research has examined effective strategies for disseminating physical activity messages, there has been no systematic effort to examine optimal message content. This paper reviews studies that evaluate the effectiveness of three approaches for constructing physical activity messages including tailoring messages to suit individual characteristics of message recipients (message tailoring), framing messages in terms of gains versus losses (message framing), and targeting messages to affect change in self-efficacy (i.e., a theoretical determinant of behavior change).

**Methods:**

We searched the MEDLINE, PsycINFO, EMBASE and CINAHL databases up to July 2008. Relevant reference lists also were searched. We included intervention trials, field experiments, and laboratory-based studies that aimed to test the efficacy or effectiveness of tailored messages, framed messages and self-efficacy change messages among healthy adults. We used a descriptive approach to analyze emerging patterns in research findings. Based on this evidence we made recommendations for practice and future research.

**Results:**

Twenty-two studies were identified. Twelve studies evaluated message tailoring. In 10 of these studies, tailored messages resulted in greater physical activity than a control message. Six studies evaluated framed messages. Five of these studies demonstrated that gain-framed messages lead to stronger intentions to be active compared to a control message. Moreover, a gain-frame advantage was evident in three of the four studies that assessed physical activity. Four studies evaluated self-efficacy change messages. The two studies that used an experimental design provide a clear indication that individuals' beliefs can be affected by messages that incorporate types of information known to be determinants of self-efficacy. Overall, strong evidence to support definitive recommendations for optimal message content and structure was lacking.

**Conclusions:**

Additional research testing the optimal content of messages used to supplement physical activity guidelines is needed. Tailored messages, gain-framed messages, and self-efficacy change messages hold promise as strategies for constructing physical activity messages and should be a focus of future research.

## Background

Physical activity guidelines for healthy adults offer evidence-based recommendations about how much physical activity it takes to reduce the risk of morbidity and mortality and/or to obtain health benefits. Guidelines are not made with the idea that they will motivate individuals to adhere to being active. Rather, they provide a general goal for telling people *how much activity to do in order to obtain benefits *(i.e., a dose-response benefit). However, in order to obtain the dose-response benefit, individuals must adhere to being regularly active over weeks, months and years of life. Thus, to motivate individuals to adhere, guidelines must be supplemented with messages that convey *why *and *how *to achieve the recommended activity level. It is through the process of messaging that the guidelines and supporting messages are disseminated to the target audience [1].

Messages and messaging are identified as critical yet distinct elements essential to the process of translating physical activity guidelines into a format appropriate for use by Canadians [1]. Messages include all of the information to be conveyed to the public (e.g., physical activity guidelines, benefits of being active, and ways to be active). Messaging is the *process *of physically getting the message to the population flowing through a medium or media (e.g., print, internet, television) that the target audience is most apt to use. It is also the process of moving the message through people to which the audience is more apt to relate, in situations that offer opportunities for action in which the audience is more apt to engage.

In the physical activity domain, the process of messaging has received the most research attention. Research examining the optimal mode (e.g., print, web-based, mass media) and the appropriate context (e.g., community-wide intervention) for message dissemination has been summarized and evaluated in a series of literature and systematic reviews [2-6]. From these reviews, it has been determined that a variety of dissemination methods including print, mass media, telephone, and online messaging *all *have potential as strategies for communicating physical activity messages. However, the impact of these strategies vary, some directly affect behavior while others are more likely to impact proximal outcomes such as individual awareness and message recall.

Another key finding from these reviews is that the likelihood of creating more enduring behavior change is maximized when messages are delivered as part of a comprehensive and multi-level behavior change intervention [3]. The VERB campaign, a well-funded, multi-faceted mass media campaign promoting physical activity for American tweens (children 9 to 13 years of age; [7]) exemplifies the impact of a comprehensive intervention approach. In this campaign, messages promoting physical activity were disseminated through mass media and school and community promotions. The messages were supported additionally by campaign partners who created opportunities (e.g., making activity spaces more accessible) for youth to be active [8]. As a result of this comprehensive approach, as message exposure increased, physical activity and positive attitudes towards physical activity increased [9] over a two-year period. The success of this campaign reinforces the conclusions drawn in existing review papers [3]. The greatest successes come when messaging is part of a larger community-based strategy in which people have multiple opportunities to be exposed to and to act on messages about physical activity.

While the existing reviews of messaging provide direction for disseminating physical activity guidelines and messages, they provide little insight into the optimal content of these messages. In essence, we know more about the process of *how *we should tell people about the guidelines than we know about *what *we should tell them [10]. It is important to examine the latter issue because the content of a message can affect the likelihood that people will pay attention to, think about, and be persuaded by the information included in a message [10,11]. Therefore, the purpose of this systematic review was to examine research testing specific message construction approaches which have the potential to inform the construction of messages that could be used to motivate people to strive toward Canada's Physical Activity Guidelines.

### The scope of the review

According to Brawley and Latimer [1], messages used to translate physical activity guidelines into practical recommendations should be salient, persuasive, and aim to change meaningful determinants of physical activity behavior. Research from the fields of health communication, marketing, and behavior change have established a variety of techniques for constructing persuasive messages [12]. Our current review examined the evidence relative to the use of three specific message construction approaches: message tailoring, message framing, and targeting messages to change self-efficacy. We focused on these three approaches because they each (a) address a critical characteristic for effective physical activity messages as outlined in our preliminary review of the literature [13], (b) could feasibly be integrated into community-wide initiatives disseminating physical activity guidelines, and (c) have a substantive body of evidence demonstrating their effects within the context of physical activity promotion. While there are a variety of other promising message construction approaches [12], there is limited evidence of their effectiveness within the physical activity domain.

First, we reviewed evidence from studies examining the impact of message tailoring on physical activity behavior. Message tailoring involves presenting information in a manner that suits the individual characteristics of the message recipient. Tailoring increases message salience [11] and the impact of the message on behavior [14]. Evaluating evidence from message tailoring research may help to determine how messages that accompany guidelines are structured and disseminated.

Second, we reviewed evidence from studies testing the impact of message framing on physical activity behavior and intentions. Message framing is the emphasis a message has on the benefits of adopting (gain-framed) or the costs of failing to adopt (loss-framed) a target behavior. In the physical activity domain, loss-framed messages emphasize the costs of being inactive (e.g., A lack of activity increases risk of diabetes) whereas gain-framed messages emphasize the benefits of being active (e.g., Get Active! Reduce your risk of diabetes). Appropriately framing health messages can enhance message persuasiveness [15]. According to message framing theory [16], using a gain-frame should optimize the persuasiveness of physical activity messages. Demonstrating the utility of gain-framed messages for promoting physical activity could have implications for revising any existing informational materials (e.g., Canada's Physical Activity Guide Canada's Physical Activity Guide to Healthy Active Living) that currently emphasize the costs of being inactive.

Finally, we reviewed strategies for constructing messages that target influential determinants of physical activity behavior. Following from the review by Rhodes and Pfaeffi in this issue [17], we opted to focus specifically on messages that aim to alter self-efficacy beliefs. According to Rhodes and Pfaeffi, self-regulatory strategies and self-efficacy beliefs have the most potential as targets for physical activity interventions compared to other theoretical constructs. The evidence for self-regulatory strategies and self-efficacy beliefs as determinants of physical activity behavior suggests that designing messages that target these constructs may have future utility. However, messaging research about the self-regulatory processes that lead to physical activity has been limited to one aspect of that process - the self-efficacy beliefs that help to encourage the use of self-regulatory skills (e.g., goal-setting; self-evaluation). Consequently, we reviewed studies that attempted to alter self-efficacy beliefs as a function of efficacy-related information included in a message promoting participation in physical activity. According to theory and practice, self-efficacy related information can be created by providing information relative to participation in a valued activity that fosters mastery experiences (e.g., successful participation/improvements in an activity), describes participation of a successful, similar-other model, provides verbal persuasion or reinforcement, and encourages monitoring of physiological and affective states. Examining self-efficacy is particularly relevant to Canada's current physical activity guides. These guides and their supporting materials contain self-efficacy related information (e.g., vignettes, persuasive messages). Evidence supporting strategies for increasing self-efficacy could affirm the utility of the message content currently used. As well, this evidence could offer additional methods for constructing future messages used to supplement current and future physical activity guidelines.

In summary, substantial research has examined effective strategies for disseminating physical activity messages. However, there has been no systematic effort to examine the optimal content of these messages. Thus, the aim of this paper was to review studies that evaluate the efficacy or effectiveness of three approaches to constructing physical activity messages including tailoring messages, gain-framing messages, and targeting messages to affect change in self-efficacy.

## Methods

### Inclusion and exclusion criteria

The general inclusion criteria for studies reviewed were: (a) the messages were communicated using minimal contact dissemination methods (e.g., brochures, videos, e-mail reminders) in which messages were delivered directly to study participants (i.e., not through mass media), (b) the primary messages encouraged physical activity only, (c) the study included a post-test message evaluation at minimum, (d) the paper was the primary report of a trial or experiment (d) the paper was written in English, (e) the study participants were healthy adults between 18-65 years of age, and (f) the outcome measures included an assessment of physical activity and/or a theoretical determinant of physical activity participation (e.g., self-efficacy).

Bauman and colleagues [18] emphasize the importance of evaluating the impact of a message using proximal (e.g., awareness), intermediate (e.g., attitudes, intentions), and distal (e.g., behavior) outcomes. Due to the nature of the research included in the review, the current review was limited to examining distal and intermediate outcomes only. Our focus on healthy adults corresponds with the target population for guideline redevelopment initiatives currently underway in Canada [19]. Moreover, it minimizes variability in study populations. There are systematic differences in demographic characteristics and determinants of physical activity participation between healthy adults and adults with a chronic disease or disability [20]. Our narrow focus on studies testing messages using minimal contact, direct-delivery dissemination methods was strategic. Researchers have greater control over the experimental manipulation in these types of studies compared to studies evaluating multi-message mass media campaigns or interpersonal communication.

Additional inclusion criteria were applied for each research question. To examine the effects of tailored messages, we included studies that used messages that were tailored to at least one characteristic of the message recipient. Because this area of research was adequately developed in that there was multiple, large randomized controlled trials, only studies with a control group were included. To examine the effects of message framing, we included studies that ascribed to the Rothman and Salovey [21] framing approach to create gain- and loss-framed messages. Gain-framed messages either emphasized the benefits attained or the costs avoided from participating in physical activity. Loss-framed messages either emphasized the costs of inactivity or the missed benefits from failing to engage in physical activity. To examine messages targeting self-efficacy, we included studies that clearly outlined how the message was constructed in order to affect this theoretical construct. Thus, studies that reported a change in self-efficacy but failed to report how the message targeted this construct were excluded (i.e., only measured efficacy and provided no detail on content).

### Identification of papers

The MEDLINE (1950 - 2008, July Week), PsycINFO (1967 - 2008, July Week 4), EMBASE (1980 - 2008 Week 30), and CINAHL (1982 - 2008 July Week 3) databases were searched. The search terms used are included in Table 1[22-43].

**Table 1:** Database search terms

General search terms	Question specific search terms
physical activity/exercise/fitness/health	1. frame/framing/gain/loss
AND	2. tailor/match/individualize
persuasion/persuasive/message/information/communication/media/education	3. self-efficacy/confidence/perceived behavioral control/competence/mastery/modeling/vicarious experience/verbal persuasion/social persuasion/feedback
	Supplemental terms^1^ 4. attitude/outcome expectancy/outcome expectancies/belief/benefit/consequence
	5. source/messenger AND credible/credibility/reliable/believable/prefer/favor/effective

*Note*. Each set of question specific search terms were use in combination with the general search terms. ^1^Supplemental search terms were included as an alternate means of identifying relevant research.

### Screening

Screening was conducted in three phases. In Phase 1, citations and abstracts were screened by a trained research assistant under the supervision of a reviewer. Papers unrelated to physical activity or that described an intervention targeting children were excluded immediately. In Phase 2, using the full set of inclusion criteria, the remaining citations and abstracts were screened by a trained research assistant and verified by a reviewer. In Phase 3, the full text of potentially relevant articles was obtained and reviewed independently by two reviewers. Discrepancies were discussed and resolved by the reviewers. The database of studies included and excluded from the review with reasons for exclusion listed is available from the first author AEL.

### Data abstraction

Two research assistants abstracted data from the articles using a standard form. Abstracted data included: sample size, baseline participant characteristics, study design, guiding theoretical framework, message characteristics (dose, format, content), outcome assessment tools, and outcome data. All abstracted data were verified independently by two reviewers.

### Criteria for assessment of study quality

A trained research assistant and a reviewer independently evaluated the methodological quality of each study. The evaluation captured elements of study and intervention design. The five criteria for assessing study design quality were based on the systematic review guidelines from the Cochrane Collaboration Back Review Group [44] and have been applied previously in a systematic review of interventions promoting physical activity. The four criteria for assessing intervention quality were derived from the evaluation schemes reported in four existing reviews of physical activity and health promotion interventions ([4,17,45,46]). The full evaluation criteria are listed in the tables included in the Additional Files. For each study, each criterion was assigned a value of 0 (no/unsure/not applicable) or 1 (yes) and a total quality score was computed (ranging from 0 to 9).

### Data analysis

We used a descriptive approach to analyze the research findings. For the message tailoring and framing studies, there was adequate data and methodological consistency to examine patterns of findings across studies. Studies that demonstrated a significant advantage for the intervention group (i.e., the tailored message in the tailoring studies or the gain-framed message in the framed message studies) versus the control group at any one assessment time point were considered to have a positive effect. Studies that demonstrated a non-significant pattern of findings that favored the intervention group were classified as having a positive trend. Due to differences in study design, the self-efficacy studies were critiqued and analyzed on an individual basis.

### Formulating practice recommendations

We formulated practice recommendations for each message construction approach. The recommendations were devised and graded based on the pre-specified process described by Tremblay and colleagues ([47]). This systematic evaluation method has been used to develop clinical practice guidelines in several domains. The evaluation provides indication of the strength of the evidence supporting a recommendation and whether the recommendations should be integrated into practice. For each recommendation, the level of evidence in favor of the message construction approach was rated on a scale from 1 (strongest evidence) to 4 (weakest evidence) using established criteria (see Tremblay et al. this issue [47,48]). Finally, the recommendation was assigned a grade of A (strong recommendation), B (intermediate recommendation), or C (weak recommendation).

## Results

### Literature search

**Figure 1:**
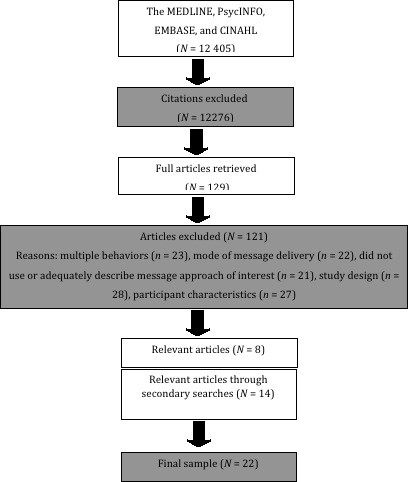
**Results of literature search**. Mode of message delivery: studies were excluded if the mode of message delivery was via mass media or required more than minimal contact; study design: studies were excluded if the study did not evaluate an appropriate outcome (i.e., physical activity, intentions or self-efficacy), the intervention was complex and prevented the isolation of message effects or no message was delivered.

The results of the literature search are depicted in Figure 1. In total, the search yielded 12,405 papers. Based on a preliminary review of article abstracts and titles, 129 papers were identified as potentially relevant and the full article was retrieved. Rigorous application of the inclusion criteria resulted in the identification of 8 studies (3 message framing studies, 3 message tailoring studies, and 2 self-efficacy studies) appropriate for review. To supplement the database search, the reference lists of the extracted articles and relevant review articles including the review by Rhodes and Pfaeffli in this issue [17] were searched resulting in the identification of an additional 14 articles (3 message framing studies, 9 message tailoring studies, and 2 self-efficacy studies). Thus, the final sample included 22 studies (6 message framing studies,12 message tailoring studies, and 4 self-efficacy studies).

### Message tailoring

#### Overview

We identified 12 [22-33]studies examining the effects of tailored messages on physical activity behavior. Details of these studies are provided in the table included in Additional File 1.

#### Message and messaging characteristics

All of the studies tailored messages to individuals' stage of change. Stages of change algorithms classify individuals into one of five stages of motivational readiness: precontemplation (i.e., no intention to become active in the next 6 months), contemplation (i.e., considering starting a physical activity regime within the next 6 months), preparation (i.e., considering becoming active in the next 30 days), action (i.e., initiation of a physical activity routine), or maintenance (i.e., participation in a physical activity routine for at least 6 months). According to the transtheoretical model [49], within each stage people vary according to their self-efficacy, their perceptions of the benefits and consequences of engaging in physical activity (i.e., decision balance) and the processes that they use to transition from one stage to the next (i.e., cognitive and behavioral processes of change). Thus, messages tailored to stages of change are designed to suit the self-efficacy, decision balance, and processes of change characteristics of each stage of motivational readiness.

In nine of the studies, messages were delivered using print media [25,31,33]. The remaining three studies [22-24,26-30,32] used websites as the mode of message delivery. Across these studies, the amount of tailored information provided varied. Four studies [23,24,30,32] provided only one dose of tailored information while eight studies delivered multiple doses of tailored messages [22,25-29,31,33].

#### Outcome measures

Self-reported physical activity behavior was the primary outcome in each of the 12 studies. In all cases behavior was evaluated using a valid and reliable assessment tool. The type of activity reported varied from leisure time physical activity (i.e., activity a person chooses to do during his or her free time; *n *= 6 [23-25,30,32,33]), lifestyle activity (i.e., activities a person engages in as part of his or her daily routine including active transport, occupational activities, and activities of daily living; *n *= 3 [23,25,33]) to total activity (i.e., any activity reported requiring energy exertion; *n *= 8 [22,23,25-29,31,33]).

#### Participant characteristics

For the most part, participants in these studies were middle- aged adult volunteers recruited from workplaces and through media advertisements. In 11 of the studies [22-25,27-33], the majority of participants were female (56 - 100% female). The activity level of participants ranged from completely inactive to regularly active and exceeding US physical activity guidelines for adults. Five studies [23,27,29,31] purposely recruited sedentary adults or individuals in the precontemplation to preparation phases. The remaining 7 studies [22,24-26,28,30,32,33] included a sample in which almost half of the participants were in the action to maintenance stages.

#### Study design features

Eleven studies [22-31,33]used a randomized controlled design and one used a non-randomized controlled design. In these studies, participants who received tailored messages were compared with participants who received no message, a message unrelated to physical activity, or a standard, generic physical activity message.

#### Methodological quality

The methodological quality of the studies varied. Seven of the studies met between 2 to 3 of the evaluation criteria. Common methodological limitations of these studies included lack of intent-to-treat analysis, failure to control for baseline activity levels in the analysis of the final outcome measure and minimal or no description of allocation concealment. While studies were clear in indicating that participants were randomized to experimental conditions, little detail regarding the method used to generate the randomization lists was given. The remaining five studies met between 4 to 7 of the criteria. Generally these studies were well-designed but had minor limitations. For example, none of the studies conducted a manipulation check to evaluate whether participants perceived that the tailored information was well-suited to their needs.

#### Summary and discussion of the evidence

A summary of tailoring effects is provided in Table 2. Overall, tailored messages resulted in significantly greater physical activity compared to a control group in more than half of the studies (7/12) [24,25,27,29-31,33]. In addition, three studies [22,26,28] demonstrated a non-significant trend or an advantage for a particular subgroup of participants (i.e., inactive participants) favoring tailored messages. Taken together, these findings suggest that message tailoring is a strategy for constructing messages to accompany physical activity guidelines that holds promise. However, because all of the studies reviewed were based upon the stages of changes, the generalizability of this message construction approach to messages tailored using other models is unknown at this time. Given that the cognitive processes proposed to underlie messages tailored to the stages of change are thought to be similar for messages tailored to other models, it is likely that effects described in the studies reviewed will hold regardless of the model to which messages are tailored [11,50].

**Table 2:** Tally of tailoring effects

Study/Message Characteristic	Significant tailoring effect	Significant and non-significant trend towards a tailoring effect
Total	58% (7/12)	83% (10/12)
Nature of the control group		
No message control/non-PA message	100% (6/6)	100% (6/6)
Generic PA message	17% (1/6)	67% (4/6)
Assessment timeframe^a^		
1 mos.	67% (2/3)	67% (2/3)
2 mos.	67% (2/3)	67% (2/3)
3 mos.	50% (2/4)	75% (3/4)
4-6 mos.	42% (3/7)	71% (5/7)
12 mos.	50% (1/2)	50% (1/2)
Mode of delivery		
Print	44% (4/9)	78% (7/9)
Online	100% (3/3)	100% (3/3)
Dose		
Single	50% (2/4)	50% (2/4)
More than 1^b^	63% (5/8)	100% (8/8)

*Note*. Twelve studies were included in the frequency analysis. ^a ^Studies with multiple assessment points are included in the tally at each relevant time point. ^b^Dose: More than 1 includes messages delivered using unlimited internet access.

It is important to consider these findings within the context of certain study design features including the type of control group and messaging strategies used. By study design, a consistent advantage (6/6) for tailored messages emerged compared to no message control group [24,25,29-31,33]. Thus providing a tailored message is better than providing no message at all. Tailored messages compared to generic physical activity message resulted in a significant effect for tailoring in one of six studies [27]. Three studies [22,26,28], however, showed promising trends. The lack of consistency in findings may be due to the nature of the generic messages. Many of the generic messages were described as having features that were appropriate to individuals in the action or maintenance stages of change. Given that a majority of the studies had approximately 40% of participants in these two phases, it may have been that the generic messages were tailored to a portion of the study sample thus confounding study findings.

By messaging approach, we consider tailoring effects in the context of mode of dissemination and dose of information on tailoring effects. In the studies reviewed, while significant tailoring effects were demonstrated using print material, these effects also were consistently demonstrated using online tools. In the one study directly comparing print and web delivery of tailored materials, it was determined that both were adequate modes of delivering a tailored message [28].

The general pattern of findings also suggests that more frequent doses of information may enhance the effects of tailored messages. Tailoring effects were reported more consistently in studies where participants received repeated exposures to tailored information than those that provided a single exposure.

### Recommendations for constructing tailored messages (Level 2, Grade B)

Overall, there is insufficient evidence to support definitive recommendations for the use of tailoring to construct messages that support physical activity guidelines (Evidence Level 2). Thus, based on the existing evidence, we suggest that it is not essential to tailor the messages that accompany physical activity guidelines. However, given that the pattern of findings indicates that tailoring messages may have some advantage over generic messages, we recommend that when the messages can be tailored easily and with little additional financial cost (e.g., messages are delivered using an online interface), tailoring should be considered. If tailoring is used, multiple exposures seem beneficial and the transtheoretical model's stages of change seem to be appropriate targets for tailoring (Grade B).

More definitively, we recommend using messages, tailored or not, to accompany physical activity guidelines. In the studies reviewed, participants who received a physical activity message consistently reported engaging in more physical activity than participants who did not receive a message or who received a general health message.

### Message framing

#### Overview

Six studies [34-39] examining the effects of gain- and loss-framed messages on physical activity behavior and/or intentions were identified. These studies are described in tables included in Additional File 2.

#### Message and messaging characteristics

As per the study inclusion criteria, all of the studies applied Rothman and Salovey [21] guidelines for framing the messages. In four of the studies, the message content was selected specifically to target theoretical determinants of physical activity (e.g., attitudes, self-efficacy). Five studies delivered the framed material using print media and one study delivered the messages via e-mail. The number of framed messages delivered varied across studies. Four studies provided only one dose of framed information while two studies provided multiple doses of framed messages.

#### Outcome measures

All of the identified studies measured participants' intentions to be active. Four studies also assessed physical activity behavior using valid and reliable self-report measures (e.g., Godin Leisure Time Exercise Questionnaire (*n *= 3; [51]; International Physical Activity Questionnaire (*n *= 1; [52])).

#### Participant characteristics

Across five of the six studies, the majority of participants were female. Participants were either undergraduate students (*n *= 4 studies) or community dwelling adults (*n *= 2 studies). Two of the studies only included participants who were sedentary. The other studies did not pre-screen participants' activity level. Thus both active and inactive participants likely were included in these studies.

#### Study design features

Two studies [37,38] used a randomized control design with pre- and post-test assessments while one study used a randomized control design with post-test assessment only [39]. In these studies, control participants received either no message whatsoever [38] or a message with a mixed frame (i.e., included both gain and loss framed information; [37,39]) analogous to standard messages currently in use. The remaining studies used a design common to message framing research that is based on the assumption that persuasive health messages naturally have either a gain- or loss-framed tone. Therefore, a no-frame control group is virtually impossible to employ and a control condition is not applicable to this design. Based on this assumption, participants in these studies were randomly assigned to either a gain or loss-framed message condition. These studies used post-test only designs. In addition to testing the main effects of framed messages, four studies examined moderated framing effects (i.e., interaction effects). One study examined message content as a moderator by comparing framed messages emphasizing either the health or self-esteem benefits of being active. Three studies examined message framing within the context of source credibility. Specifically, the effects of message framing were compared across messages which were conveyed to participants from sources with high (e.g., doctor) versus low (e.g., student) credibility.

#### Methodological quality

The methodological quality of the studies varied. The variation is not surprising. Many of the studies were conducted as lab-based, proof-of-principle experiments. As such, these experiments were not designed to meet standard quality criteria for randomized controlled intervention trials. For example, two of the studies [34,39] did not have a follow-up assessment making it impossible to conduct intent-to-treat analyses. Thus, when we applied our criteria for methodological quality which were based largely on criteria for randomized controlled trials, four of the studies received scores between 1 and 3. For these studies, the methodological limitations of greatest concern were the absence of a theoretical framework to guide message content, a lack of pilot testing and the failure to conduct an evaluation of message use or processing. The other two studies received scores ranging from 4-6. The studies were well-designed but had some minor limitations such as a lack of intent-to-treat analyses. Once again, the lack of intent-to-treat analyses reflects the fact that the studies were designed as efficacy rather effectiveness trials.

#### Summary and discussion of the evidence

The effect of framed messages on physical activity are discussed first, for their effects on behavior and second, for their effects on intentions. Of the four studies that assessed the effects of framed messages on physical activity behavior, three reported framing effects. In the study by Latimer and colleagues [37], the sedentary adults who received three gain-framed messages reported more physical activity than those who received loss-framed and standard use, mixed-framed messages. In the study by Parrott and colleagues [38], sedentary participants who received gain-framed messages reported more physical activity compared participants in the no message control condition. The gain-framed messages also led to greater participation in physical activity compared to the loss framed messages, however, only among participants who had low baseline activity levels of physical activity. A moderated framing effect also was reported in the study by Jones and colleagues [35]. When messages came from a highly credible source, gain-framed messages evoked greater physical activity participation than loss-framed messages. Framing effects were not observed when the message source had low credibility [36]. With three out of four studies reporting an advantage favoring gain-framed messaged, these findings suggest that when delivering messages from a credible source such as the Public Health Agency of Canada to adults who are inactive, gain-framed messages may be more advantageous than loss-framed and standard-use, mixed frame messages.

All of the framing studies reviewed included an assessment of intentions. Intentions are considered a direct determinant of physical activity behavior [53] and thus an important target for physical activity messages. Overall, framing effects were reported in five of the studies; two studies reported a main effect and three studies reported a moderated effect. The two main-effect only studies both favored the gain-framed message compared to a loss-framed message [37,38]. Also, the gain-framed message was superior to a no message control [38]. Gain-framed messages had no advantage compared to a mixed-framed message [37,39] when considering their effects on intentions.

In studies examining moderated framing effects, three studies found that under certain conditions, gain-framed messages resulted in stronger intentions to be active compared to a loss-framed message [34,35,39] or no message [39]. One study [36] found no effects whatsoever. In the study by Robberson and Rogers [39] comparing messages targeting the self-esteem benefits versus the health benefits of physical activity, a gain- framed advantage emerged only when messages targeted self-esteem. In the studies examining the moderating effects of source credibility, although a gain-framed advantage emerged, the nature of the interaction was inconsistent. Arora and colleagues [34] found that only when the message was attributed to a low credibility source, gain-framed messages evoked stronger physical activity intentions than loss-framed messages. Conversely, Jones and colleagues [35] found that only when messages came from a highly credible source, gain-framed messages evoked stronger physical activity intentions than loss-framed messages. Moreover, in a replication of their 2003 study, Jones and colleagues [36] did not find evidence of message source as moderator. The equivocal pattern of findings across the studies examining message source as a moderator suggests that there is insufficient evidence to determine the optimal conditions for delivering gain-framed messages. Nonetheless, it is clear that message source should be considered when delivering framed-messages.

Although unrelated to message framing per se, there is a finding from all three studies that examined source credibility that warrants mention. There was a consistent advantage for messages delivered from a highly credible source, regardless of message frame. Messages attributed to a highly credible source led to stronger intention and greater physical activity participation (when assessed) than messages attributed to low credible sources. This pattern of findings is wholly consistent with evidence from communications research on a variety of topics that has repeatedly demonstrated the importance of delivering messages through a credible source (e.g., [54]).

### Recommendations for constructing framed messages (Level 2, Grade B)

The pattern of main and moderated effects of framed messages on physical activity behavior and intentions seems sufficiently consistent (Evidence Level 2) to cautiously recommend the use of gain-framed messages rather than loss-framed messages for creating messages to accompany physical activity guidelines (Grade B). Some research has begun to examine the utility of mixed-framed messages. The findings have been equivocal. Until further evidence is available, it seems prudent to use strictly gain-framed messages to encourage physical activity participation (Grade B).

### Messages targeting self-efficacy

#### Overview

We identified two proof-of principle-type experiments, a field experiment, and a randomized controlled trial that met the inclusion criteria for this review. Our overview of these four studies addresses issues related to the study outcomes and design first because these matters provide conceptual background for discussing all other aspects of the study. These studies are described in the tables included in Additional File 3.

#### Outcome measures

In each study the outcomes on which we focused for this review were combined measures of perceived behavioral control and self-efficacy. Given that perceived behavioral control and self-efficacy are two constructs that are part of *different *theories but are considered to be conceptually similar by some researchers, conceptual definitions are important to understand in order to orient the reader.

Perceived behavioral control (PBC) is defined as individuals' expectancy that performance of physical activity and related behavior is within their control. Behavioral control can be broadly described as ranging from easily performed behaviors to behavioral goals needing specialized skills, opportunities, and human and physical resources. Judgments of perceived behavioral control are assumed to take into account both an individual's internal personal resources and external influences that would affect execution of a behavior [55].

Self-efficacy is defined as individuals' beliefs about their abilities to coordinate those skills and abilities to secure the goals they want in specific circumstances or domains (e.g., physical health, education). Bandura [56] also emphasizes that it is individuals' beliefs in their ability to "organize and execute the courses of action required to produce given attainments" (p.3). Self-efficacy theory [56] presents self-efficacy beliefs as a central variable in the theory with direct effects upon behavior assuming that individuals have sufficient incentive (e.g., desired goals).

Although some theorists argue that these constructs are similar, there is sufficient evidence to suggest that, depending on measurement, perceived behavioral control is different from self-efficacy [57]. The former concept concerns both the ease and difficulty of performing a behavior (i.e., for me starting exercise is easy.....difficult) *and *individuals' perception of whether performance of the behavior is actually up to them (i.e., starting exercise is mostly up to me; if I want to exercise, it is mainly up to me). The latter, self-efficacy, focuses on individuals' beliefs about taking courses of action (i.e., their specific confidence) that bring about their specific goals (e.g., I am x% confident in my ability to....... "schedule/plan to bring about regular exercise"; "regroup/revise my plans to adapt to unplanned change or obstacles and still exercise").

As per the operational definitions described above, the three experimental studies targeted change in perceived behavioral control/self-efficacy as one of their outcome variables. The randomized controlled trial used change in self-efficacy as an outcome measure.

#### Study design features

The study design features varied. The two proof of principle-type experiments [40,43] used a between groups comparison design where individuals read separate written messages with content hypothesized to vary the reaction to reading the message. In these experiments, participants who received a message meant to bolster perceived behavioral control/self-efficacy (high efficacy messages) were compared with participants who received messages meant to inhibit perceived behavioral control/self-efficacy (low efficacy messages). The third study was a field experiment [41]. In the main experimental treatment group, participants viewed a DVD designed to affect theoretical determinants of physical activity behavior change including self-efficacy. Participants were randomized to view either the experimental video or an attention control video about cancer and nutrition information or they were randomized to a no-contact control condition. The fourth study [42] was a randomized controlled trial. The trial was carefully designed using child care centers stratified for levels of socioeconomic status. Individuals within centers were randomly assigned to a control condition, a print materials only condition, or a print plus discussion group condition. The print materials provided self-efficacy information. Thus the print only group was our primary group of interest. However, in the study analyses the reference group was the print plus group. Participants in this group received the print materials, engaged in a group of possible local community strategies to promote physical activity (e.g., community-based support from fitness leadership, other exercising mothers, support from relationship partner) and received a telephone call to reaffirm discussion information and a notice board for information sharing/support.

#### Message and messaging characteristics

These studies exposed participants to messages containing information that concerned the self-management of physical activity. The two proof of principles experiments and the randomized controlled trial delivered their messages in print form. The field experiment delivered the message in DVD format. All studies only delivered the message for one exposure. In the three experiments, message content was guided primarily by the protection motivation theory (PMT [58]). The framework guiding the content of the messages included in the randomized controlled trial was not specified. For our purposes, the information presented relative to perceived behavioral control/self-efficacy is described herein.

Participants in the experimental investigation by Stanley and Maddux [43] read a description of a new exercise program followed by a unique element. Participants assigned to the high efficacy condition received a message outlining a new exercise program that was easy to follow and complete, with 95% of participants completing the program. By contrast, those individuals receiving the low efficacy message read that most people were unable to complete the new exercise program and that there were a large percentage of drop-outs from those who had been involved. Thus, the efficacy-related information suggested that the mastery of the program was either easier with individuals persisting in the program or hard resulting in people leaving the program.

Using a similar experimental paradigm as Stanley and Maddux [43], the study by Courneya and Hellston [40] provided students with messages concerned with exercise and colon cancer risk and included unique elements. The low perceived behavioral control message emphasized 5 to 6 days per week of high intensity exercise for one hour as reducing risk in contrast to the high perceived behavioral control message which suggested only 2 to 3 days per week of moderate exercise. Thus, the efficacy-related information described an exercise prescription that was complex and difficult to control or less complex and easy to control.

In the field experiment, the experimental video concerned colon cancer and exercise. Self-efficacy/perceived behavioral control information focused upon the how-to-do aspects of planning and incorporating physical activity and exercise into lifestyle in order to accumulate 30 minutes of moderate to vigorous physical activity daily. In the randomized controlled trial, the print materials focused on physical activity benefits and ways to overcome barriers to physical activity. The overcoming barriers portion of the print materials targeted information that participants presumably could use to adapt to barriers that interfered with being regularly active.

#### Participant characteristics

The two experimental studies used young healthy university undergraduates as their participants. The field experiment that used the DVD format for message delivery used middle-aged school employees. The randomized controlled trial used women with young children. Two of the three experimental investigations reported samples of greater than 70 percent females. The randomized controlled trial was exclusively female.

#### Methodological quality

All four studies used a randomized design. The first two experiments satisfied 4 of the 9 quality criteria. These studies were conducted as proof-of-principle experiments and not designed to meet quality criteria for randomized controlled intervention trials. Thus, some quality criteria were either not applicable or because of information not being provided, evaluation of the criterion was uncertain. The field experiment and randomized controlled trial exposed participants to multi-component messages and thus sacrificed testing "pure " message effects on any one theoretical component in favor of an overall theory effect or combined multiple component effect. These latter two studies assessed the individual self-efficacy variable in their evaluation but any change would have to be attributed to the overall message versus the self-efficacy message content alone. These studies contrast with the experiments in that regard. Although participants in the experiments read all messages based upon components of the PMT, these messages were read separately and their effects analyzed for independent influence. While it could be argued that the reading of all messages could have overlapping influence and thus no pure effects of one PMT variable could be detected, this influence was not like the influence of the completely blended messages in the field experiment and RCT. The two experiments were able to consider the main effects analysis of each variable in the overall experiment. Where the analysis detected a main effect of the self-efficacy/perceived behavioral control variable, there was no interaction effect of the other PMT variables. Thus the evidence for the influence of the persuasive message was strongest in the two proof-of-principles experiments.

#### Summary and discussion of the evidence

The two experimental studies provide a clear indication that when individuals read structured messages that incorporate types of information known to be determinants of self-efficacy/perceived behavioral control, study participants' beliefs can be affected. In the study by Stanley and Maddux [43] perceived control/self-efficacy was greater when participants read messages outlining a new exercise program that was easy to follow and complete (high efficacy message) compared to when participants read messages indicating that most people found the new exercise program difficult and were likely to drop out (low efficacy message). The study by Courneya and Hellston [40] found that participants receiving the less complex exercise prescription expressed significantly more control than participants in the more complex exercise prescription group. The studies provide some promise that messages can be successfully tailored to impact on beliefs known to be related to future exercise intentions and exercise behavior in a variety of predictive studies (e.g., [55,56].

Compared to the two experimental studies, the findings from the field experiment and the randomized controlled trial provide less indication of how message content influences efficacy-related beliefs. In the field experiment by Graham and colleagues [41], the message delivered by DVD was of influence on selected variables, but was ineffective for altering PBC. The measure of PBC did not correspond to the how-to-do information presented in the video and this may have been a reason that the message failed to have an effect on the perceived behavioral control/self-efficacy outcome. In the randomized controlled trial [42], the residual change in self-efficacy in the print materials plus discussion/support group (Condition 3) was in a positive direction compared to the other conditions, the effect was not significant. Moreover, the print media alone distribution of information about overcoming physical activity barriers was insufficient to stimulate a change in self-efficacy compared to the more complex Condition 3 intervention.

### Recommendations for constructing messages to change self-efficacy (Level 3, Grade C)

The findings of the 4 studies using messages to change self-efficacy/perceived behavioral control are mixed. There is insufficient evidence to confirm a reliable systematic effect (Level 3). However, a closer look reveals that when the messages are theory-based, carefully controlled and the content is targeted to specifically influence the dependent variable using determinants or conditions known to alter beliefs about efficacy and perceived control, the results are somewhat more promising. The first two studies we reported in this section used this specifically-targeted approach. The caveats related to the results of these two experiments is that they are limited to educated, undergraduate university students and the actual influence of altering these control beliefs on actions related to physical activity behavior (e.g., first steps initiating activity, enrolling in a fitness class, obtaining fitness advice) is unknown (Grade C).

## Discussion

The purpose of this systematic review was to examine the evidence testing the utility of three message construction approaches (tailored messages, framed messages, and self-efficacy change messages) that could be used to inform the content of messages that accompany physical activity guidelines. Twenty-two studies were reviewed. Overall, we could *not *conclude that there was strong evidence to support definitive recommendations for optimal message content. Rather, findings from this review point to several promising practices as summarized in Table 3. We cautiously advocate the use of message tailoring only when it can be done when with ease and with little extra cost. We also suggest that it seems advisable to use gain-framed message when possible. Finally, we recommend the provision of information known to influence determinants known to alter beliefs about efficacy and control as a potential strategy for constructing messages aiming to boost self-efficacy. It is important to consider these recommendations in light of existing research and limitations of the review.

**Table 3:** Summary of recommendations for practice

		Evaluation	
		
	Recommendation	Level	Grade
General Recommendation	We recommend using messages to encourage physical activity participation as set out by physical activity guidelines.		
Message Tailoring	Tailoring messages may have some advantage over generic message, we recommend that when the medium for dissemination is suitable (e.g., delivered online), tailoring should be considered. If tailoring is used, multiple exposures seem beneficial.	2	B
Message Framing	Messages accompanying physical activity guidelines should be gain-framed messages rather than loss-framed messages. Until further evidence is available, it seems prudent to use strictly gain-framed messages to encourage physical activity participation rather than mixed-framed	2	B
Self-Efficacy Change Messages	To construct self-efficacy enhancing messages, the use of theory-based, carefully controlled and designed to specifically influence determinants or conditions known to alter beliefs about efficacy and control is a strategy that holds promise and should be considered.	3	C

In our review of message tailoring research, a pattern of findings emerged that favored tailored messages compared to generic messages or no message whatsoever. Tailoring effects tended to emerge more consistently with multiple message exposure. The findings from our review align with message tailoring research in the broader health domain. For example, a meta-analysis [14] of 57 studies testing tailored messages promoting a variety of health behaviors (e.g., practicing safe sex, consuming more fruits and vegetables) found a small albeit significant, message-tailoring advantage. Moreover, the effects reported in the Noar meta-analysis tended to be larger when there was exposure to multiple messages. The consistency between the findings from our review of physical activity studies and the findings from the broader health behavior change literature support the notion that message tailoring should be considered a promising practice with potential to enhance the effectiveness of the messages accompanying physical activity guidelines.

Our review of the message-framing research, also recommends that message framing is a sufficient and promising practice, but not essential. The pattern of findings for behavioral and social-cognitive outcomes in the studies we reviewed was consistent with the theory-based hypothesis that gain-framed would be more persuasive than loss- or mixed-frame messages. Our suggestion that framing physical activity messages has potential to enhance message effectiveness differs from the conclusion drawn from a meta-analysis [59] of 93 studies examining the effects of framed health messages. This meta-analysis found a small but significant advantage for gain-framed over loss-framed messages for encouraging disease prevention behaviors such as dental hygiene, physical activity, and healthy eating. However, when the data were examined by behavior type, a significant gain-framed advantage was only apparent for dental hygiene behaviors. In their analysis of physical activity messages specifically, the effect size was small (*r *= .11, CI: -.056, .270) and approached, but did not reach standard levels of significance. This analysis was underpowered and differed from our review in two ways. First, our review did not include all of the same studies as the meta-analysis. We included two additional, recently published studies with large sample sizes, multiple message exposure, and results favoring gain-framed messages. We also considered moderated framing effects. This approach of examining moderators is consistent with an emerging research direction that is aiming to specify more precisely when gain-framed messages will be most effective [60]. Given these fundamental differences, it is not surprising that our conclusion differs from earlier research.

Our review of messages meeting our criteria for selection and targeting change in self-efficacy beliefs as one outcome yielded only four studies; two proof-of-principles experiments, one field experiment and one complex intervention. The lack of systematic research testing physical activity messages that target theoretical determinants such as self-efficacy is disappointing. In 1987, Olson and Zanna [61] suggested that physical activity messaging could be improved by utilizing a theoretical foundation to design the content of messages. Evidently, few researchers have heeded this call.

Given the evidence gaps in this research area, our recommendation for including information known to affect recipients' self-efficacy beliefs is really only a suggestion for constructing future messages associated with physical activity guidelines. There is insufficient evidence to make a definitive recommendation. Researchers and message designers would be wise to consider research from the counselling-based intervention literature in both symptomatic and asymptomatic populations that have proven effective in altering self-efficacy and other theoretical determinants of physical activity behavior (e.g., [62]). Consideration of this literature may generate ideas for research that will fill the gap in the physical activity message construction literature with respect to self-efficacy.

### Quality of evidence

Our cautious suggestions for the use of tailored, framed, and self-efficacy change messages were due in part to the limited quality of the evidence. In a standard assessment of methodological quality based on criteria for randomized controlled intervention trials, the message framing and tailoring studies fared poorly. The self-efficacy studies also were subject to several design limitations. These gaps in methodology are reflective of the relatively immature state of the research area. For example, the application of message tailoring and message framing principles to physical activity promotion has only slowly emerged over the last decade. As the field has matured, the methodological rigour in the most recent studies has increased steadily (refer to tables summarizing quality assessment in Additional Files). However, it should also be recognized that identified methodological gaps are partly reflective of the differing approaches to experimental design across multiple fields (i.e., Psychology, Health Communications, Health Promotion) rather than gaps that might normally be associated with a single field and one design paradigm. The judgment of insufficient evidence to make definitive recommendations is appropriate when the standard of evaluation is the randomized clinical trial. However, a number of the effects we identified were observed in experimental studies and we have confidence that these studies met quality standards at a level commensurate with peer-reviewed proof-of-principle studies for their respective fields. Thus, we have made suggestions for the future rather than recommendations per se.

Two notable limitations of the majority of studies in the review were the sole reliance on self-report measures to assess physical activity and the predominance of women in the study samples. Although the studies reviewed used valid and reliable measures of physical activity, the measures were constructed primarily for use in much larger cohort studies and as result tend to lack sensitivity to small changes in behavior [63]. Also, the measures assessed different behaviors (e.g., leisure time physical activity versus lifestyle activity) which were not always consistent with the behavior promoted in the message. Future research should reconsider the type of behavioral measure expected to change as a function of the message. For example, if small changes in volume of activity (i.e., frequency x minutes) are expected as a function of a persuasive message, then using an objective measure of physical activity (e.g., accelerometer) may detect small, consistent change. However, if a message is expected to change a proximal or intermediate outcome (e.g., consulting with a fitness professional; downloading physical activity suggestions from the internet; signing up for a "try-out fitness class; or reading supplemental material from Canada's Physical Activity Guide to Healthy Active Living) rather than physical activity, then new behavioral measures are needed to assess message effectiveness.

The second limitation, the large proportion of women in the reviewed studies, limits the external validity and any generalizability of findings to the population at large. Health promotion research in general lacks evidence of effective strategies for changing men's behavior [64] and future research on this population segment is clearly required.

In addition to these general limitations, a specific limitation of the tailoring studies was the narrow scope of tailoring approaches used. All studies tailored messages using the stages of change. Research related to other health behaviors (e.g., healthy eating) has demonstrated the utility of tailoring to a variety of demographic [65] and personality characteristics [50]. These alternate tailoring methods hold promise and should be examined in future physical activity messaging research.

### Quality of the review

Our systematic review had several limitations. A database search strategy different than what we used is needed for future systematic reviews. The small yield from our database search in part is a reflection of the field being underdeveloped. As a result, there are relatively few studies to be included in the review regardless of the search strategy. Also, the investigators who conduct message evaluation research are from a variety of fields. For example, psychologists, physical activity scientists, and communication experts differ in what they mean by the term social marketing. Keywords from these fields and literatures vary widely thereby limiting their systematic application in a database search.

An additional limitation was our exclusion of studies conducted among clinical populations (e.g., patients with diabetes). The common mandate for the evidence-based reviews for physical activity guidelines in this issue, including our review, was a focus on research concerning healthy adult participants. Although there is a need to promote healthy lifestyle practices in clinical populations, and there is promising research identifying characteristics of effective messages and strategies for these individuals, this was necessarily excluded from our review.

Finally, our review focused primarily on intermediate (e.g., theoretical determinants) and distal outcomes (e.g., behavior change). This specific focus was necessary given the nature of the research included in the review - few studies included proximal outcomes (e.g., awareness). Among the studies that assessed proximal outcomes the measurement approach varied across studies precluding meaningful comparisons. Although proximal outcomes were not considered in the current review, they should be included in future studies evaluating message effectiveness. These outcomes are important indicators of message and campaign success [18].

### Recommendations for future research

We were unable to make definitive recommendations for practice given that there was insufficient evidence. The small amount of systematic research examining the appropriate content for physical activity messages unquestionably highlights the need for additional research in this area. However, we can make general recommendations for future research examining optimal message content for constructing persuasive messages for physical activity. These pertain to the three approaches we reviewed and are as follows:

• More controlled experimental research is needed to isolate effective message characteristics and examine the generalizability of study findings. These studies should be carefully designed to minimize confounders such as the mode of message delivery. Specifically, we do not know whether presentation by a combination of modalities enhances or detracts from individuals' attention. Thus, it is important to reliably determine the effects of the content of the messages before advancing to more complex multi-method modes of message presentation.

• Research also should examine the optimal dose of information required to maximize message effectiveness.

Our recommendations for future investigation specific to each of the message construction approaches are as follows:

#### Message tailoring

• Additional research is needed among sedentary adults (i.e., an optimal target group for physical activity messages) comparing tailored messages to mismatched messages *and *generic physical activity messages in the same study. This study design provides a rigorous test of tailored messages (c.f. Blissmer and colleagues [22]).

• Research should begin to test the impact of messages that are tailored to characteristics other than message recipients' stages of change (e.g., using different theoretical foundations and determinants of physical activity). Several studies in the broader realm of health promotion have begun to establish the effectiveness of messages tailored to psychological constructs that differentiate people on the basis of how they process incoming health information (e.g., [50]). A practical advantage of this approach is that messages are tailored to *stable *dispositional characteristics. Thus, for example, the need for repeated assessments in stage of change studies of messages tailored to dynamic message recipient characteristics is eliminated.

#### Message framing

• Additional large, multi-message randomized controlled trials targeting inactive adults are needed to strengthen the evidence-base regarding use of gain-framed messages to promote physical activity participation.

• Researchers should continue to investigate factors that moderate framing effects. This research helps to refine and advance current message framing postulates by specifying more precisely *when *gain- and loss-framed messages will be most effective [60].

#### Messages targeting self-efficacy

• Conduct more randomized control experiments to demonstrate the effectiveness of altering sources of efficacy-relevant information in targeted messages to change efficacy beliefs and behavior.

• Researchers must carefully select outcome measures appropriate to the goal of the message. To gauge message effectiveness accurately, the measurement of self-efficacy/perceived control beliefs should correspond to the message content. Appropriate indicators (i.e., new measures) of behaviors to be changed must also be reconsidered. Probable behaviors most likely to be affected by a persuasive message relate to behavioral "first steps" toward physical activity. Examples of these behavioral steps *are not necessarily *immediate and regular types of activity participation, but rather, immediate, motivated actions such as getting further information; developing plans; and enrolling in an activity.

## Conclusions

To motivate individuals in a behavioral direction that may lead to future adherence to regular physical activity, guidelines must be supplemented with messages that convey not only *what individuals should do *but also *why *and *how *they should do it. Research examining the optimal content of messages encouraging physical activity is an emerging field. To date, there is insufficient evidence from the three message construction approaches we reviewed to lead to definitive, practical recommendations for persuasive messages that would support the dissemination of physical activity guidelines. We suggest that the effects of message tailoring, message framing, and targeting sources of self-efficacy enhancing information show promise but more research systematically isolating effective message characteristics is required.

## Abbreviations

Note. Most abbreviations appear in the tables included in the Additional Files. PA: physical activity; SE: self-efficacy; RE: Response Efficacy; PV: Perceived Vulnerability; PS: Perceived Severity; PMT: Protection Motivation Theory.

## Competing interests

This work was funded by the Public Health Agency of Canada (PHAC) and the authors received an honorarium from PHAC for completing the systematic review. AEL and LRB have also received honoraria and speaker fees from several other non-for profit organizations that have an interest in physical activity and health.

## Authors' contributions

AEL directed and was involved in all aspects of this systematic review including conceptualization, data abstraction, and manuscript preparation. LRB participated in the design of the research questions and manuscript preparation. RLB contributed to the development of the research questions and the literature review and data abstraction process. RLB also helped to draft the manuscript. All authors read and approved the final manuscript.

## Supplementary Material

Additional file 1 **Message tailoring - Quality assessment and study summary tables**. The tables summarize the results of the assessment of study quality and describe the research methods and the results of the message tailoring studies. Click here for file

Additional file 2 **Message framing - Quality assessment and study summary tables**. The tables summarize the results of the assessment of study quality and describe the research methods and the results of the message framing studies. Click here for file

Additional file 3 **Self-efficacy change messages - Quality assessment and study summary tables**. The tables summarize the results of the assessment of study quality and describe the research methods and the results of the studies evaluating self-efficacy change messages. Click here for file
