# Supplementary material for: A systematic review of three approaches for constructing physical activity messages: What messages work and what improvements are needed?
Source: Int J Behav Nutr Phys Act. 2010 May 11;7:36. doi: 10.1186/1479-5868-7-36 (PMC2885311; doi:10.1186/1479-5868-7-36)
Supplement: Additional file 1 — Message tailoring - Quality assessment and study summary tables. The tables summarize the results of the assessment of study quality and describe the research methods and the results of the message tailoring studies. [file 1479-5868-7-36-S1.DOC]

**Quality assessment of message tailoring studies**

|  | Blissmer & McAuley [22] | Bull et al [23] | Cardinal & Sachs [24] | Hager et al [25] | Marcus, Emmons et al [26] | Marcus, Bock et al [27] | Marcus, Lewis et al [28] | Marcus, Napolitano et al [29] | Marshall et al. [30] | Napolitano et al [31] | Naylor et al [32] | Spittaels et al [33] |
| --- | --- | --- | --- | --- | --- | --- | --- | --- | --- | --- | --- | --- |
| Theoretical framework used to guide message content cited | yes | yes | yes | yes | yes | yes | yes | yes | yes | yes | yes | yes |
| Messages were pilot tested prior to use | yes | no | no | no | yes | yes | no | yes | yes | no | yes | yes |
| Effects of message manipulation verified | no | no | no | no | no | no | no | no | no | no | no | no |
| The use of messages was reported | no | yes | no | yes | no | no | yes | no | yes | yes | no | yes |
| Was randomization described | yes | yes | yes | no | no | no | yes | yes | yes | no | yes | yes |
| Allocation concealment | unsure | unsure | unsure | unsure | unsure | unsure | yes | unsure | yes | unsure | no | unsure |
| Outcome assessment independent and blind | unsure | unsure | unsure | unsure | unsure | unsure | unsure | unsure | yes | unsure | unsure | unsure |
| Final outcome measure controlled for baseline physical activity | no | no | no | no | no | no | yes | no | no | yes | no | no |
| Intent-to-treat analysis used | no | no | no | no | no | no | yes | yes | yes | no | no | yes |
| Total | 3 | 3 | 2 | 2 | 2 | 2 | 6 | 4 | 7 | 3 | 4 | 5 |

*Note. Unsure* means that this quality criterion was not addressed in the study report. This may be a function of type of study, journal reporting requirements and differences between proof-of-principle experiments and randomized controlled trials.

**Summary of message tailoring studies with focus on behavior change**

| **Study** | **Sample** | **Design** | **Tailoring approach** | **Messaging** | **Outcome**  **variables** | **Findings** |
| --- | --- | --- | --- | --- | --- | --- |
| Blissmer & McAuley [22] | *N* = 196 employees  *M* age = 43.4 (*SD*=10.9)  74% female  44% action/ maintenance | RCT (comparison: mismatched, standard PA message, non-PA standard message control)  Follow-up: 4 mos. with stage reassessed every mos. | TTM  Mismatched: same as tailored but participants only receive materials that differed from their current stage | Format: Print (mail)  Dose: 4 (1/mos.) | PA: Aerobic Center Longitudinal Study PA Questionnaire [66] total energy expenditure | 4 mos. No significant difference between groups in total PA or lifestyle PA. Tailored and standard PA messages resulted in moderate effect size increase in lifestyle PA |
| Bull et al. [23] | *N*=272 adults in the contemplation or preparation stage  *M* age = 49 (*SD* = 16.4)  74% female | RCT (comparison: general and personalized (GAP), general and not personalized (GNP), no message control)  Follow-up: 3 mos. | TTM, exercise goal, PA motives and barriers, and preferred type of PA | Format: Print (mail)  Dose: 1 (3 days post baseline) | PA: number of days/wk participated in at least 30 min of LTPA, ADL, and total PA | No difference in PA |
| Cardinal & Sachs [24] | *N* = 113 adults  *M* age = 36.9 (*SD*=not given)  100% female  39.0% in action/ maintenance | RCT with 2 tailored message groups (comparison: standard health information message)  Follow-up: 1 mos. | TTM, SCT  Group 1: promoted small increases in PA through ADL plus tailored messages  Group 2: promoted standard recommendations plus tailored messages | Format: Print (mail)  Dose: 1 | PA: Godin Leisure Time Exercise Questionnaire [51] Total LTPA | 1mos. Lifestyle PA + tailored message group reported more PA than control. No difference between, standard recommendation + tailored messages and other groups. |

| **Study** | **Sample** | **Design** | **Tailoring approach** | **Messaging** | **Outcome**  **variables** | **Findings** |
| --- | --- | --- | --- | --- | --- | --- |
| Marcus, Emmons et al [26] | *N*=1559 employees from 11 worksites  *M* age = 39.9 (*SD*=10.9)  43% female  14% in action stage | RCT (comparison: standard PA messages)  Follow-up: 3 mos. | TTM | Format: Print (mail)  Dose: 2 (baseline & 1 mos.) | PA: 7-day PA recall [67] | No group x time effect on PA.  Group x stage interaction indicated that at follow-up the tailored group at all stages except maintenance reported more min. of PA than control. |
| Marcus, Bock et al [27] | N=194 sedentary adults (not engaging in 30 min. mod. PA at least 5 d./wk)  M age = 44.3 (SD=10.8)  76.3% female | RCT (comparison: standard message control)  Follow-up: 1, 3 & 6 mos. | TTM, SCT | Format: Print (Mail)  Dose: 3 (baseline, 1, & 3 mos. (matched to next stage)) | PA: 7-day recall [67] Total activity (light to vigorous) | 1 mos. no group x time effects  3 mos. Tailored group more likely to meet PA recommendations  6 mos. Tailored group reported more min. of PA and were more likely to meet PA recommendations |
| Marcus, Lewis et al [28] | *N*=249 sedentary adults (<90 mins of PA each week)  *M* age = 44.5 (*SD*=9.3)  83.7% female | RCT with two tailored groups (comparison: standard website control)  Follow-up: 6 & 12 mos. | TTM, SCT | Format: Group 1: Print; Group 2: Internet  Dose: Group 1: monthly tailored feedback; Group 2: unlimited web access with monthly tailored feedback | PA: 7-day recall [68]; [67] Total activity (light to vigorous) | 6 & 12 mos. No differences between group in total PA, fitness, or likelihood of meeting PA recommendations. Non-significant trend at 6 mos. in total PA favors tailored groups. |
| Marcus, Napolitano et al [29] | *N* = 239 sedentary adults (≤90 min of mod. – vig. PA/wk)  *M* age = 44.5 (*SD*=9.3)  82% female | RCT with 2 tailored message groups (comparison: non-PA standard message)  Follow-up: 6 & 12 mos. | TTM | Format: Group 1: Print (mail); Group 2: telephone  Dose:14 contacts | PA: 7-day recall [68]Total activity (light to vigorous) | 6 mos. Greater increase in PA. No difference between tailored groups.    12 mos. Greater increase in PA in tailored print than other groups. No difference between tailored phone and control in PA. |

| **Study** | ***Sample*** | **Design** | **Tailoring approach** | **Messaging** | **Outcome**  **variables** | **Findings** |
| --- | --- | --- | --- | --- | --- | --- |
| Marshall et al [30] | *N*=462 adults  *M* age = 49.0 (*SD*=5.7)  57% female  11% in action phase | RCT (comparison: no message control)  Follow-up: 2 & 6 mos. | TTM | Format: Print (mail)  Dose: 1  Received booklets corresponding to current stage and all higher stages | PA: 14-day recall [69,70] walking and moderate-to-vigorous LTPA | 2 mos. Tailored group reported more min. of PA and more likely to meet PA recommendations than control.  6 mos. No between group differences |
| Napolitano et al [31] | *N*=65 sedentary adults (≤120 min. mod. PA or ≤60 min. vig. PA/week)  *M* age = 42.8 (*SD*=10.0)  86% female | RCT (comparison: no message control)  Follow-up: 1 & 3 mos. | TTM, SCT | Format: Website and e-mail  Dose: Unlimited internet access  12-weekly e-mail tip sheets | PA: BRFSS [71] mod. min & walking | 1 mos. Tailored group reported more min. of mod PA and walking than control.  3 mos. Tailored group reported more min. of walking. |
| Naylor et al [32] | *N*=294 adults  *M* age = 42.4 (*SD*=15.1)  77% female  18.4% in action/ maintenance | Quasi-experimental , non-randomized, clustered by primary care practices (comparison: 5 min. PA counselling, tailored material & 5 min. PA counselling)    Follow-up: 2 & 6 mos. | TTM | Format: Print (face-to-face)  Dose: 1 (following baseline) | PA: Activity Assessment Questionnaire [72] Total activity (light to vigorous) | 2 mos. & 6 mos. No group x time interactions emerged for PA, stage of change, or SE |

| **Study** | **Sample** | **Design** | **Tailoring approach** | **Messaging** | **Outcome**  **variables** | **Findings** |
| --- | --- | --- | --- | --- | --- | --- |
| Spittaels et al [33] | *N* = 434 adults  *M* age = 41.4 (*SD*=5.6)  66.1% female  43.5% compliance with PA recommendations | RCT with 2 tailored groups (comparison: no message control)  Follow-up: 6 mos. | TTM, Theory of Planned Behavior | Format: Website  Dose: Group 1: Unlimited website access, 1 tailored feedback form; Group 2: same as Group 1 plus 7 non-tailored e-mail prompts & 1 additional tailored feedback form | PA: Long International Physical Activity Questionnaire [52] LTPA, sitting, active transport, total moderate-vigorous PA  Other: information use (read, discussed, saved) | 6 mos. Both tailoring groups reported significant increase in LTPA and active transportation and significant decreases time spent sitting compared to control. No differences in amount of PA between tailoring groups Significant increase in proportion of multiple tailoring group (Group 2) who met PA recommendations at follow-up than other groups. |

*Note.* PA = Physical activity

**References**

22. Blissmer B, McAuley E: **Testing the requirements of stages of physical activity among adults: the comparative effectiveness of stage-matched, mismatched, standard care, and control interventions.** *Ann Behav Med* 2002, **24:**181-189.

23. Bull FC, Kreuter MW, Scharff DP: **Effects of tailored, personalized and general health messages on physical activity.** *Patient Educ Couns* 1999, **36:**181-192.

24. Cardinal BJ, Sachs ML: **Effects of mail-mediated, stage-matched exercise behavior change strategies on female adults' leisure-time exercise behavior.** *J Sports Med Phys Fitness* 1996, **36:**100-107.

25. Hager RL, Hardy A, Aldana SG, George JD: **Evaluation of an internet, stage-based physical activity intervention.** *American Journal of Health Education* 2002, **33:**329-336.

26. Marcus BH, Emmons KM, Simkin-Silverman LR, Linnan LA, Taylor ER, Bock BC, Roberts MB, Rossi JS, Abrams DB: **Evaluation of motivationally tailored vs. standard self-help physical activity interventions at the workplace.** *Am J Health Promot* 1998, **12:**246-253.

27. Marcus BH, Bock BC, Pinto BM, Forsyth LH, Roberts MB, Traficante RM: **Efficacy of an individualized, motivationally-tailored physical activity intervention.** *Ann Behav Med* 1998, **20:**174-180.

28. Marcus BH, Lewis BA, Williams DM, Dunsiger S, Jakicic JM, Whiteley JA, Albrecht AE, Napolitano MA, Bock BC, Tate DF et al.: **A comparison of internet and print-based physical activity interventions.** *Arch Intern Med* 2007, **167:**944-949.

29. Marcus BH, Napolitano MA, King AC, Lewis BA, Whiteley JA, Albrecht A, Parisi A, Bock B, Pinto B, Sciamanna C et al.: **Telephone versus print delivery of an individualized motivationally tailored physical activity intervention: Project STRIDE.** *Health Psychol* 2007, **26:**401-409.

30. Marshall AL, Bauman AE, Owen N, Booth ML, Crawford D, Marcus BH: **Population-based randomized controlled trial of a stage-targeted physical activity intervention.** *Ann Behav Med* 2003, **25:**194-202.

31. Napolitano MA, Fotheringham M, Tate DF, Sciamanna C, Leslie ER, Owen N, Bauman A, Marcus BH: **Evaluation of an internet-based physical activity intervention: a preliminary investigation.** *Ann Behav Med* 2003, **25:**92-99.

32. Naylor PJ, Simmonds G, Riddoch C, Velleman G, Turton P: **Comparison of stage-matched and unmatched interventions to promote exercise behaviour in the primary care setting.** *Health Educ Res* 1999, **14:**653-666.

33. Spittaels H, De B, I, Vandelanotte C: **Evaluation of a website-delivered computer-tailored intervention for increasing physical activity in the general population.** *Prev Med* 2007, **44:**209-217.

66. Kohl HW, Blair SN, Paffenbarger RS, Jr., Macera CA, Kronenfeld JJ: **A mail survey of physical activity habits as related to measured physical fitness.** *Am J Epidemiol* 1988, **127:**1228-1239.

67. Sallis JF, Haskell WL, Wood PD, Fortmann SP, Rogers T, Blair SN, Paffenbarger RS, Jr.: **Physical activity assessment methodology in the Five-City Project.** *Am J Epidemiol* 1985, **121:**91-106.

68. Blair SN, Haskell WL, Ho P, Paffenbarger RS, Jr., Vranizan KM, Farquhar JW, Wood PD: **Assessment of habitual physical activity by a seven-day recall in a community survey and controlled experiments.** *Am J Epidemiol* 1985, **122:**794-804.

69. Booth ML, Owen N, Bauman A, Gore CJ: **Relationship between a 14-day recall measure of leisure-time physical activity and a submaximal test of physical work capacity in a population sample of Australian adults.** *Res Q Exerc Sport* 1996, **67:**221-227.

70. Booth ML, Owen N, Bauman AE, Gore CJ: **Retest reliability of recall measures of leisure-time physical activity in Australian adults.** *Int J Epidemiol* 1996, **25:**153-159.

71. Jones DA, Ainsworth BE, Macera CA: **Reliability and validity of walking questions in the Behavioral Risk Factor Surveillance System (BRFSS).** *Medicine and Science in Sports and Exercise* 1999, **31:**S232.

72. Riddoch C, Murphy N, Nichols A, van Wersche A, Cran G: **Report of the Northern Ireland Health Survey.** *Queen's University of Belfast, Belfast* 1990.
