# Supplementary material for: A systematic review of three approaches for constructing physical activity messages: What messages work and what improvements are needed?
Source: Int J Behav Nutr Phys Act. 2010 May 11;7:36. doi: 10.1186/1479-5868-7-36 (PMC2885311; doi:10.1186/1479-5868-7-36)
Supplement: Additional file 2 — Message framing - Quality assessment and study summary tables. The tables summarize the results of the assessment of study quality and describe the research methods and the results of the message framing studies. [file 1479-5868-7-36-S2.DOC]

Quality assessment of message framing studies

|  | Arora et al. [34] | Jones, Sinclair, Courneya [35] | Jones, Sinclair et al [36] | Latimer et al [37] | Parrott et al. [38] | Robberson & Rogers [39] |
| --- | --- | --- | --- | --- | --- | --- |
| Theoretical framework used to guide message content cited | no | no | yes | yes | yes | yes |
| Messages were pilot tested prior to use | no | no | yes | yes | no | no |
| Effects of message manipulation verified | yes | yes | yes | yes | no | yes |
| The use of messages was reported | no | yes | yes | no | no | no |
| Was randomization described | no | no | no | yes | no | no |
| Allocation concealment | unsure | unsure | unsure | yes | no | unsure |
| Outcome assessment independent and blind | unsure | unsure | unsure | unsure | unsure | unsure |
| Final outcome measure controlled for baseline physical activity | N/A | yes | no | yes | yes | N/A |
| Intent-to- treat analysis used | N/A | no | no | no | yes | N/A |
| Total | 1 | 3 | 4 | 6 | 3 | 2 |

*Note. Unsure* means that this quality criterion was not addressed in the study report. This may be a function of type of study, journal reporting requirements and differences between proof of principle experiments and randomized controlled trials. *Not applicable* (N/A) means that intent to treat was not a consideration given the short-term effects of exposure and zero attrition from message exposure. Corresponding references:

**Summary of message framing studies**

|  | **Sample** | **Design** | **Messaging** | **Outcome variable** | **Findings** |
| --- | --- | --- | --- | --- | --- |
| Arora et al. [34] | *N*=136 community residents  *M* age = not stated (*SD*=not stated)  55% female  Stage not stated | Randomized Experiment  Frame: gain or loss  Source: high or low credibility | Format: Print (newsletter)  Dose: 1 | Intentions: 3-items | Main Effect: None:  Moderated Effect: When message from low-credibility source gain > loss  for intentions |
|  |  |  |
| Jones, Sinclair, Courneya [35] | *N*=192 undergraduates  *M* age = 19.81 (*SD*=4.05)  72% female  Stage not stated | Randomized experiment  Frame: gain or loss  Source: high or low credibility or no source | Format: Print (pamphlet)  Dose: 1 | Behavior: GLTEQ [51] Intentions: 3-items [73] | Main Effect: None  Moderated Effect: When messages from credible source: Gain>loss for intentions and physical activity |
| Jones, Sinclair et al. [36] | *N*=450 first year undergraduates  *M* age = 20.02 (*SD*=3.94)  70% female  Stage not stated | Randomized experiment  Frame: Gain or loss  Source: Credible, non-credible or no source | Format: Print (pamphlet)  Dose: 1 | Behavior: GLTEQ [51] Intentions: 3-items [74] | Main Effect: None  Moderated Effect: None |
| Latimer et al.  [37] | *N*=322 callers to the U.S. National Cancer Institute Cancer Information Service  *M* age = 47.4 (*SD*=12.03)  76% female  0% action phase (all sedentary) | RCT  Frame: gain, loss or mix | Format: Telephone, print  Dose: 4 | Behavior: IPAQ [52]Intentions: level of agreement with statement “I intend to participate in regular PA over the next 2 weeks” [75] | Main Effect: Gain>Loss and mixed for physical activity behavior. Gain > loss for intentions  Moderated Effect: None |
| Parrott et al. [38] | *N*=170 undergraduates  *M* age = 20.2 (*SD*=0.9)  38% female  100% not meeting ACSM guidelines for PA (30 min. of mod.-vig. Intensity >5 d/wk.) | Pre-post  Frame: gain, loss or none | Format: E-mail  Dose: 2 weeks, every other day | Behavior: GLTEQ [51]  Intentions: “Over the next two weeks, I intend to exercise ___ times per week” [76] | Main Effect: gain > loss for intentions  Moderated Effect: Dependent on baseline level behavior: Gain > loss and control when baseline behavior <1.2 |
| Robberson & Rogers [39] | *N*= 84  undergraduates  *M* age = not stated (*SD*=not stated)  100% female  0% action phase (all sedentary) | RCT  Frame: gain or loss  Message target: health or self-esteem | Format: Print (essay)  Dose: 1 | Intentions: 4-items | Main Effect: None  Moderated Effect: When messages appeal to self-esteem: Gain or mixed >loss for intentions. When messages appeal to health for loss>mixed for: Intentions |

*Note.* GLTEQ = Godin Leisure Time Exercise Questionnaire, IPAQ = Long International Physical Activity Questionnaire.

**References**

34. Arora R, Stoner C, Arora A: **Using framing and credibility to incorporate exercise and fitness in individuals' lifestyle.** *J Consum Market* 2006, **23:**199-207.

35. Jones LW, Sinclair RC, Courneya KS: **The effects of source credibility and message framing on exercise intentions, behaviors, and attitudes: an integration of the elaboration likelihood model and prospect theory.** *J Appl Soc Psychol* 2003, **33:**179-196.

36. Jones LW, Sinclair RC, Rhodes RE, Courneya KS: **Promoting exercise behaviour: an integration of persuasion theories and the theory of planned behaviour.** *Br J Health Psychol* 2004, **9:**505-521.

37. Latimer AE, Rench TA, Rivers SE, Katulak NA, Materese SA, Cadmus L, Hicks A, Hodorowski JK, Salovey P: **Promoting participation in physical activity using framed messages: an application of prospect theory.** *Br J Health Psychol,* **13:** 659-681.

38. Parrott MW, Tennant LK, Olejnik S, Poudevigne MS: **Theory of planned behavior: Implications for an email-based physical activity intervention.** *Psychology of Sport and Exercise* 2008, **9:**511-526.

39. Robberson MR, Rogers RW: **Beyond fear appeals: negative and positive persuasive appeals to health and self-esteem.** *J Appl Soc Pscyhol* 1988, **18:**277-287.

51. Godin G, Shephard RJ: **A simple method to assess exercise behavior in the community.** *Can J Appl Sport Sci* 1985, **10:**141-146.

52. Craig CL, Marshall AL, Sjostrom M, Bauman AE, Booth ML, Ainsworth BE, Pratt M, Ekelund U, Yngve A, Sallis JF et al.: **International physical activity questionnaire: 12-country reliability and validity.** *Med Sci Sport Exerc* 2003, **35:**1381-1395.

73. Courneya KS: **Predicting repeated behavior from intention - the issue of scale correspondence.** *J Appl Soc Psychol* 1994, **24:**580-594.

74. Courneya KS: **Understanding readiness for regular physical activity in older individuals: an application of the theory of planned behavior.** *Health Psychol* 1995, **14:**80-87.

75. Armitage CJ, Conner M: **Distinguishing perceptions of control from self-efficacy: Predicting consumption of a low-fat diet using the theory of planned behavior.** *J Appl Soc Psych* 1999, **29:**72-90.

76. Rhodes RE, Courneya KS: **Threshold assessment of attitude, subjective norm, and perceived behavioral control for predicting exercise intention and behavior.** *Psychol Sport Exerc* 2005, **6:**349-361.
