# Supplementary material for: A systematic review of three approaches for constructing physical activity messages: What messages work and what improvements are needed?
Source: Int J Behav Nutr Phys Act. 2010 May 11;7:36. doi: 10.1186/1479-5868-7-36 (PMC2885311; doi:10.1186/1479-5868-7-36)
Supplement: Additional file 3 — Self-efficacy change messages - Quality assessment and study summary tables. The tables summarize the results of the assessment of study quality and describe the research methods and the results of the studies evaluating self-efficacy change messages. [file 1479-5868-7-36-S3.DOC]

Quality assessment of self-efficacy studies

|  | Courneya & Hellston [40] | Graham et al [41] | Miller et al [42] | Stanley & Maddux[43] |
| --- | --- | --- | --- | --- |
| Theoretical framework used to guide message content cited | yes | yes | no | yes |
| Messages were pilot tested prior to use | no | yes | no | no |
| Effects of message manipulation verified | yes | no | no | yes |
| The use of messages was reported | yes | no | no | yes |
| Was randomization described | yes | yes | yes | yes |
| Allocation concealment | unsure | unsure | unsure | unsure |
| Outcome assessment independent and blind | unsure | unsure | unsure | unsure |
| Final outcome measure controlled for baseline physical activity | no | no | no | no |
| Intent-to- treat analysis used | N/A | N/A | no | N/A |
| Total | 4 | 3 | 1 | 4 |

*Note. Unsure* means that this quality criterion was not addressed in the study report. This may be a function of type of study, journal reporting requirements and differences between proof-of-principle experiments and randomized controlled trials. *Not applicable (N/A)* means that intent to treat was not a consideration given the short term effects of exposure and zero attrition from message exposure

Summary of self-efficacy studies

| **Study** | **Sample** | **Design** | **Self-Efficacy Message Content** | **Messaging** | **Outcome**  **variables** | **Findings** |
| --- | --- | --- | --- | --- | --- | --- |
| Courneya & Hellston [40] | *N*=427 undergraduates  *M* age = 19.7 (SD=4.0)  73% female  Stage not stated | Randomized factorial experiment --  2 (hi/lo SE) x 2(hi/lo RE) x 2(hi/lo perceived vulnerability (PV))x 2(hi/lo perceived severity (PS))  Follow-up: immediately post message | Theoretical Framework: Protection Motivation Theory (SE, RE, PV, and PS manipulated)  SE: described the amount of PA needed to reduce risk of cancer (hi SE: 2-3 d./wk for 20 min. mod intensity; lo SE: 5-6 d./wk for 60 min. high intensity. | Format: Print (essay)  Dose: 1 essay (1 message/theoretical construct) | SE: perceived behavioral control (three 7-point items; [53]) | SE: Compared to lo SE message, hi SE message led to greater SE |
| Graham et al [41] | N=72 school employees  M age = 43.81 (SD=11.50)  70% female  100% precontemplation to preparation stages | RCT with PMT message group (2 comparison groups: non PA message, no message)  Follow-up: baseline, immediately post message, 2 weeks, 4 weeks | Theoretical framework: Protection Motivation Theory (SE, RE, PV, and PS manipulated)  SE: ways to integrate more PA into daily routine, write goals and put reminders around the house | Format: DVD  Dose: 1 video (20 min) | SE: perceived behavioral control (four 7-point items; [53]) | SE: no difference in SE between groups; planned comparisons revealed that the PMT message group had greater SE than the control groups |

| **Study** | **Sample** | **Design** | **Self-Efficacy Message Content** | **Messaging** | **Outcome**  **variables** | **Findings** |
| --- | --- | --- | --- | --- | --- | --- |
| Miller et al [42] | *N* = 554 women with children  *M* age = 33.1 (SD=4.4)  100% female  Stage not stated | RCT with print message group (2 comparison groups: no message control, print message plus barrier related discussion groups and community intervention)  Follow-up: baseline and 8 and 28 weeks post baseline | Theoretical framework: Social cognitive theory (SE and RE manipulated)  SE: described strategies to overcome barrier PA relevant to mother | Format: print (mail)  Dose: 1 booklet | SE: barrier SE [77] | SE: no difference between groups in SE residual change. |
| Stanley & Maddux [43] | *N* = 195 undergraduates (not enrolled in an exercise program similar to the one described in the message)  *M* age = not stated (*SD*=not stated)  % female not stated | Randomized factorial experiment --  2 (hi/lo self-efficacy (SE)) x 2 (hi/lo response efficacy (RE)) x 2 (hi/lo outcome value (OV))  Follow-up: immediately post message | Theoretical Framework: Protection Motivation Theory and Self-Efficacy (SE, RE, OV manipulated)  SE essay: the ease (hi SE) or difficulty (lo SE) of completing an PA program | Format: Print (essay)  Dose: 3 essays (1 essay/ theoretical construct) | SE: perceived ease/difficulty of completing a PA program | SE: Compared to lo SE message, hi SE message led to greater SE and intentions. Hi SE tended to be more likely to sign up than lo SE. |

*Note.*  PA = physical activity, SE = self-efficacy, RE = Response Efficacy, PV = Perceived Vulnerability, PS = Perceived Severity, PMT = Protection Motivation Theory

**References**

40. Courneya KS, Hellsten LAM: **Cancer prevention as a source of exercise motivation: an experimental test using protection motivation theory.** *Psychol Health Med* 2001, **6:**59-64.

41. Graham SP, Prapavessis H, Cameron LD: **Colon cancer information as a source of exercise motivation.** *Psychology and Health* 2006, **21:**739-755.

42. Miller YD, Trost SG, Brown WJ: **Mediators of physical activity behavior change among women with young children.** *Am J Prev Med* 2002, **23:**98-103.

43. Stanley MA, Maddux JE: **Cognitive processes in health enhancement: investigation of a combined protection motivation and self-efficacy model.** *Basic and Applied Social Psychology* 1986, **7:**101-113.

53. Ajzen I: **The theory of planned behavior.** *Organizational Behavior and Human Decision Processes* 1991, **50:**179-211.

77. Marcus BH, Owen N: **Motivational readiness, self-efficacy and decision-making for exercise.** *J Appl Soc Psychol* 1992, **22:**3-16.
